# Alcohol Consumption Before Myocardial Infarction

**Published:** 1995

**Authors:** Michael H. Criqui

**Affiliations:** Michael H. Criqui, M.D., M.P.H., is a professor in the Departments of Family and Preventive Medicine and director, Preventive Cardiology Academic Award, University of California at San Diego, San Diego, California

**Keywords:** AOD consumption, myocardial infarction, therapeutic dry effect, moderate AOD use

**Figure:**
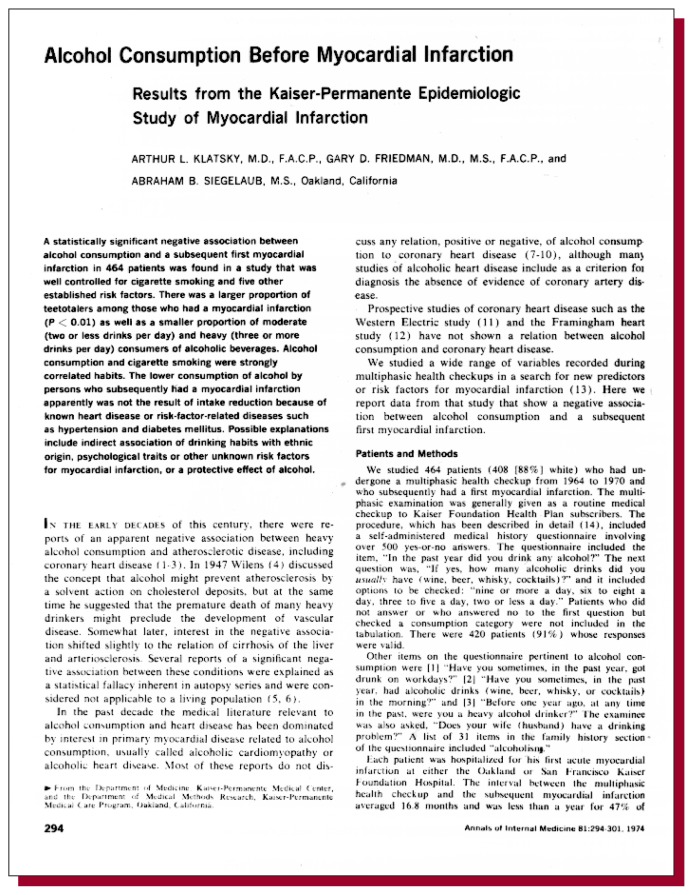
Klatsky, A.L.; Friedman, G.D.; and Siegelaub, A.B. Alcohol consumption before myocardial infarction. Results from the Kaiser-Permanente epidemiologic study of myocardial infarction. *Annals of Internal Medicine* 81:294–301, 1974.

Klatsky and colleagues published their seminal article on alcohol’s effects on the health of the heart more than 20 years ago (Klatsky et al. 1974). Although suggestions had been made in the early part of this century that alcohol might have some beneficial effect on the prevention of atherosclerotic diseases such as heart attacks, researchers in 1974 probably were cynical about such claims for at least two reasons. First, studies reporting that patients with cirrhosis of the liver, a disease often associated with heavy alcohol consumption, showed lower-than-normal incidence of atherosclerotic disease had been dismissed as a statistical artifact. Second, two epidemiologic studies, one in Chicago, IL (1963), and one in Framingham, MA (1966), had reported no significant association between alcohol consumption and subsequent heart attacks.

Klatsky and colleagues found that among the group of 464 patients they studied, which included abstainers, moderate drinkers, and heavy drinkers, the abstainers had the highest proportion of heart attacks. This provocative finding apparently resulted from exploring a long list of risk factors in a search for “new predictors . . . for myocardial infarction” (i.e., heart attack). Having found strong statistical support for a theory discredited by the Chicago and Framingham studies, the authors were admirably cautious. Their discussion should serve as a model for other investigators. They addressed seven separate possible sources of bias in the data as well as the possibility that this result might be attributable to any single type of alcohol rather than all alcoholic beverages. Finally, they discussed the plausibility of a biological effect of alcohol on coronary circulation.

How could Klatsky and colleagues find a highly significant association between alcohol consumption and lower incidence of heart disease in a case-control study when two prospective studies had not? Apparently neither the Chicago nor the Framingham study had taken into consideration the strong correlation of alcohol consumption with cigarette smoking. Because cigarette smoking is a powerful risk factor for heart attack, smokers present in the earlier studies would have caused any beneficial effect of alcohol to be underestimated. Complex statistical techniques such as multivariate risk models, which are routinely employed today to adjust for the potentially confounding affects of multiple variables (such as cigarette smoking), were still in their early development in 1974. Klatsky and colleagues clearly realized that these other variables could obscure alcohol’s effects. Thus, for each case, they employed not only an “ordinary” control, but a “risk” control. Although by definition neither control had had a heart attack, only the risk control was matched with study patients for risk of heart attack on several factors, including the key variable of smoking, thus minimizing the potential for confounding.

The researchers’ discussion of possible bias in the results seems prescient in retrospect. They presented data showing no evidence that subjects at risk for heart disease had voluntarily reduced or stopped their alcohol consumption. A later study argued that such a bias was present (Shaper 1990). However, other studies, which carefully explored whether subjects who were at high risk of heart attack had voluntarily become abstainers, found little evidence to support Shaper’s hypothesis and confirmed Klatsky and colleagues’ earlier findings (Klatsky et al. 1986; Criqui 1990; Jackson et al. 1991; Rimm et al. 1991).

In 1974 it was unclear why alcohol might afford some protection against heart attack. The authors addressed the plausibility of a protective effect at the end of the discussion but had little to say on the subject. Their focus understandably was on cardiac physiology, and there was no clear evidence from cardiac physiology to explain alcohol’s potentially beneficial effect on the heart. It was well known then, as now, that heavy alcohol consumption could damage the heart muscle and produce a specific condition called alcoholic cardiomyopathy.

The landmark paper by Klatsky and colleagues helped to stimulate careful evaluation of the association between alcohol consumption and heart disease in several large-scale, long-term epidemiological studies. We have learned much in the two decades since. We now know that the increase in high-density lipoprotein (HDL) cholesterol, which accompanies moderate alcohol consumption, may be responsible for one-half or more of moderate amounts of alcohol’s beneficial effect on the incidence of heart disease (Criqui et al. 1987; Langer et al. 1992). We also know that moderate consumption may have an effect through reduced blood clotting (Meade et al. 1979; Renaud et al. 1992). However, we also have clearly learned the limit of any alcohol benefit to the heart. Only moderate drinkers (i.e., those who consume one to two drinks per day) gain any overall health advantage (Bofetta et al. 1990, Criqui 1994). Higher consumption levels may result in increases in stroke and other cardiovascular diseases, some cancers, cirrhosis, and accidents and violence. In fact, the optimal benefit against heart attack is achieved at one to five drinks per week, or less than one drink per day (Gronbaek et al. 1994).

As additional research on alcohol and heart disease continues to unfold, Klatsky and his colleagues are not resting on their laurels. They continue to publish careful, reasoned contributions on this topic from studies conducted with the Northern California Kaiser health maintenance organization population. Such studies include the differential effects of age, ethnicity, and beverage preference as well as the overall effects of alcohol consumption on alcohol-related disease and death (Klatsky et al. 1992; Klatsky et al. 1993).
